# β-Hydroxybutyrate Alleviates Atherosclerotic Calcification by Inhibiting Endoplasmic Reticulum Stress-Mediated Apoptosis via AMPK/Nrf2 Pathway

**DOI:** 10.3390/nu17010111

**Published:** 2024-12-30

**Authors:** Yu Chen, Yiran You, Xin Wang, Yufeng Jin, Yupeng Zeng, Zhijun Pan, Dan Li, Wenhua Ling

**Affiliations:** 1 Department of Nutrition, School of Public Health, Sun Yat-Sen University, Guangzhou 510080, China; 17343611467@163.com (Y.C.); youyr@mail2.sysu.edu.cn (Y.Y.); wangx523@mail2.sysu.edu.cn (X.W.); jinyf6@mail2.sysu.edu.cn (Y.J.); zengyp27@mail2.sysu.edu.cn (Y.Z.); panzhj5@mail2.sysu.edu.cn (Z.P.); lidan58@mail.sysu.edu.cn (D.L.); 2 Guangdong Provincial Key Laboratory of Food, Nutrition and Health, Guangzhou 510080, China; 3 School of Public Health and Management, Ningxia Medical University, Yinchuan 750101, China

**Keywords:** β-Hydroxybutyrate, atherosclerotic calcification, endoplasmic reticulum stress, apoptosis

## Abstract

Background: Atherosclerotic calcification (AC) is a common feature of atherosclerotic cardiovascular disease. β-Hydroxybutyrate (BHB) has been identified as a molecule that influences cardiovascular disease. However, whether BHB can influence AC is still unknown. Methods and Results: In this study, ApoE^−/−^ mice, fed a Western diet, were used to examine the effects of BHB on AC. Rat vascular smooth muscle cells (VSMCs) were used to verify the impacts of BHB on AC and to explore the underlying mechanisms. The results show that Western diet-challenged ApoE^−/−^ mice, supplemented with BHB for 24 weeks, exhibited reduced calcified areas, calcium content, and alkaline phosphatase (ALP) activity in the aortas, as well as ameliorated severity of AC. Furthermore, BHB downregulated the expression of glucose-regulated protein 78 (GRP78) and C/EBP homologous protein (CHOP), thereby reducing endoplasmic reticulum stress (ERS) and ERS-mediated apoptosis in the aortas of the mice. Consistently, in vitro studies showed that BHB reduced ALP activity and calcium content in VSMCs, and inhibited VSMC calcification. Additionally, BHB suppressed ERS-mediated apoptosis in VSMCs. Conclusions: In summary, the present results demonstrate that BHB can alleviate atherosclerotic calcification by inhibiting ERS-mediated apoptosis. Therefore, BHB may serve as a viable therapeutic agent for AC.

## 1. Introduction

Atherosclerosis is a prevalent cardiovascular disease that accounts for a major proportion of all-cause mortality worldwide [1]. It is a chronic pathological process characterized by inflammation, accumulation of lipids, and intimal calcification in the large arteries [2]. The rupture of atherosclerotic plaque will result in severe cardiovascular events [3]. Moreover, atherosclerotic calcification (AC), caused by the formation of calcium phosphate crystals in the intimal vessel wall, can impair vascular function and influence the plaque stability [4]. In particular, plaques containing microfractures and fragmented calcifications are susceptible to rupture, leading to thrombosis and vascular occlusion [5]. Therefore, inhibiting the development of AC may help reduce atherosclerosis-related cardiovascular complications.

The osteochondrogenic transition of vascular smooth muscle cells (VSMCs), characterized by upregulation of osteogenic phenotype markers such as runt-related transcription factor 2 (RUNX2) and downregulation of the contractile protein α-smooth muscle actin (α-SMA), plays a crucial role in the formation of calcification within atherosclerotic lesions [6,7]. Most osteochondrogenic precursor-like cells in atherosclerotic lesions are derived from VSMCs [5]. VSMC apoptosis can promote plaque calcification, as apoptotic bodies released from VSMCs serve as the original nucleation sites for the growth of calcium-phosphate crystals [6,8]. Therefore, reducing VSMC apoptosis may be a useful strategy for AC prevention.

Multiple factors promote the formation of atherosclerosis, including hyperlipidemia, oxidative stress, and inflammation, all of which also induce endoplasmic reticulum stress (ERS) in VSMCs [9,10]. ERS can trigger VSMC apoptosis and contribute to their mineralization and osteogenic transformation, leading to the calcification of the vessel wall and aortic valves [11,12]. Thus, inhibiting ERS-mediated apoptosis may be a promising strategy to delay the development of AC. The adenosine monophosphate-activated protein kinase (AMPK) has been identified as a critical cellular energy sensor and a vital regulator of metabolic homeostasis [13]. Additionally, the nuclear factor erythroid 2-related factor 2 (Nrf2) is an essential transcription factor for maintaining cardiovascular health [14]. AMPK mediates the antioxidative cascade by activating Nrf2 [15], and the AMPK/Nrf2 pathway is involved in maintaining endoplasmic reticulum homeostasis [16] and the pathological progression of atherosclerosis [17]. However, the role of the pathway AMPK/Nrf2 in regulating the progress of AC remains unclear.

In recent years, the beneficial effects of ketone body β-Hydroxybutyrate (BHB) have been increasingly recognized [18]. Apart from serving as a substitute energy source when blood glucose levels are low, emerging evidence has shown that BHB is also an important regulatory molecule that exerts protective effects against heart failure [19], hypertension [20], atherosclerosis [21], and medial arterial calcification [22]. However, variations in dosage and frequency of BHB supplementation have led to inconsistent results. The duration of BHB intervention in previous studies has varied from several hours to 10 weeks, and studies involving prolonged BHB supplementation are lacking [22,23,24]. Although one study demonstrated that BHB could inhibit medial arterial calcification in chronic kidney disease [25], the mechanisms governing medial and intimal arterial calcification, including atherosclerotic calcification, remain unclear. Another study reported that BHB could ameliorate atherosclerosis by maintaining endoplasmic reticulum homeostasis, but the supplementation lasted only 10 weeks, and the effects of BHB on ERS-mediated apoptosis were not identified [23]. In summary, the effects of a prolonged BHB supplementation on the calcification progress of atherosclerosis and its associated risk factors are still unclear. Therefore, this study aims to clarify the effects of 24 weeks of BHB supplementation on AC and identify the potential underlying mechanisms.

A widely used AC mouse model [26] and rat VSMC were adopted in this study. We found that BHB can mitigate AC in the model mice by suppressing ERS-mediated apoptosis of VSMCs and improving the blood glucose and lipid profiles of mice. This study suggests that long-term BHB supplementation may be a potential approach for preventing and treating AC.

## 2. Materials and Methods

### 2.1. Animal Experiments

Male ApoE^−/−^ mice, aged 8 weeks, were obtained from Medicience (Biomedicine, Yangzhou, China) and fed with a Western diet (21% fat and 0.15% cholesterol) (MD12015, Biomedicine, China) for 24 weeks to induce atherosclerotic calcification. The mice were housed in a pathogen-free facility at a temperature of 25 ± 2 °C with a 12:12 h light/dark cycle, providing unrestricted access to food and water. All mice were randomly assigned to three groups (n = 6–8): ordinary chow diet (CD) + PBS; Western diet (WD) + PBS; and WD + BHB (166898, Sigma, St. Louis, MO, USA) (100 mg/kg, gavage, daily). Mice in the CD and WD groups were gavaged with an equal volume of PBS. The BHB treatment lasted for 24 weeks. Body weight and food intake were recorded weekly. Oral glucose tolerance tests were conducted at the beginning, middle, and end of the experiment. At the end of this study, all animals were fasted for 12 h, then euthanized using sodium pentobarbital (200 mg/kg, i.p.). After drawing blood samples, cold PBS was injected via the left ventricle to perfuse the mice. The heart was thoroughly dissected with the aorta, the aortic root was promptly embedded in an OCT compound and frozen for future section-making and staining operations, while the aorta was stored at −80 °C for Western blot analysis, calcium content measurement, and ALP activity assays. All procedures were approved by the Institutional Animal Care and Use Ethics Committee at Sun Yat-sen University (No. SYSU-SPH-2020-013, approval date: 11 March 2020) and performed under the criteria of the National Institutes of Health (NIH) Guide for the Care and Use of Laboratory Animals.

### 2.2. Cell Experiments

Rat VSMCs were purchased from ScienCell Research Laboratories (Carlsbad, CA, USA). The cells were cultured at 37 °C with 5% CO_2_ in Dulbecco’s modified Eagle medium (DMEM), supplemented with 10% fetal bovine serum, which served as a growth medium (GM). To induce VSMC calcification, the growth medium was supplemented with 10 mM beta-glycerophosphate (Sigma, G9422-10G) and 3 mmol/L CaCl_2_ (Sigma, 21097-50G), referred to as the calcification medium (CM). To assess the effect of BHB on cell calcification, VCMCs were exposed to varying concentrations of BHB (1, 2, and 4 mM) for 7 days in the presence of CM. The medium was changed every two to three days, and cells from passages 3 to 8 were used. The calcium deposition, ALP activity, and calcium content of cells were measured. Western blot analysis and cell apoptosis staining were performed. Each experiment was repeated four times.

### 2.3. Blood Ketone Measurement

To ensure that ketosis was induced by BHB gavage, blood ketone levels were measured before (time 0) and after gavage (30, 60, 90, and 120 min) using tail blood samples and a Freestyle Optium Neo (Abbott, IL, USA).

### 2.4. Oral Glucose Tolerance Test

The oral glucose tolerance test (OGTT) was performed at the beginning (week 0), the middle (week 12), and the end (week 24) of the intervention. After 12 h of fasting, blood was collected from the tail vein to measure the fasting blood glucose using a portable glucometer (Abbott, IL, USA). Mice were then gavaged with 1 g/kg glucose, and blood glucose levels were measured at 15, 30, 60, 90, and 120 min post-gavage.

### 2.5. Blood Lipid Profiles Analysis

The blood of mice was collected after 12 h of starvation. Serum lipid profiles, including total cholesterol (TC), high-density lipoprotein cholesterol (HDL-C), low-density lipoprotein cholesterol (LDL-C), and total triglyceride (TG) were measured using enzymatic assay kits (Nanjing Jiancheng, Nanjing, China).

### 2.6. Alizarin Red S Staining of Aortic Roots and VSMC

Aortic roots in OCT were cut into 8 µm cryostat sections, spanning from the point where the three aortic valves first appeared to where they disappeared. The frozen sections were then returned to room temperature, fixed, and incubated with Alizarin Red S (Beyotime, Shanghai, China) for 30 min. Excess dye was then rinsed off three times with deionized water. For VSMCs, cells were washed with PBS, fixed with 4% paraformaldehyde at room temperature, and then stained with Alizarin Red S for 5 min. Images were captured using an inverted microscope (Leica DMI4000B, Leica, Wetzlar, Germany). The staining areas were quantified by Image J software version 1.8.0 (NIH, Bethesda, MD, USA).

### 2.7. Determination of Calcium Content of Aortas and VSMC

The descending aortas were freeze-dried to a constant weight and then soaked in 0.6 mM HCl for 48 h at 37 °C to extract calcium. Calcium contents were detected using a calcium assay kit (BioAssay System, Hayward, CA, USA). The calcium levels were calculated through colorimetric analysis, normalized to tissue dry weight, and expressed as mM/mg. For VSMCs, cells were washed and decalcified in 0.6 M HCl for 30 min. The supernatants were taken to measure the calcium content. After that, a solution containing 0.1 M NaOH and 0.1% SDS was used to solubilize cells, and then the cell protein concentrations were determined with a protein assay kit (Thermo Fisher Scientific, Waltham, MA, USA). The cell calcium content was expressed as mM/mg protein after being corrected to the protein concentration.

### 2.8. Determination of ALP Activity of Aortas and VSMC

ALP activity was measured using a commercial kit (Jiancheng Biology Engineering Institute, Nanjing, China). In short, aortic tissues or cells were homogenized at 4 °C. The homogenates were used for protein content and ALP activity measurements. The ALP activity of each sample was normalized to the protein concentration.

### 2.9. TUNEL Staining in Aortic Roots and VSMC

Apoptosis in aortic roots and VSMCs was evaluated using a terminal deoxyribonucleotidyl transferase-mediated dUTP-digoxigenin nick-end labeling (TUNEL) staining kit (Solarbio Science, Beijing, China). Briefly, aortic root sections or VSMCs were returned to room temperature, fixed, permeabilized, and then incubated with the TUNEL reaction buffer. Cell nuclei were counterstained with DAPI buffer (Phygene Life Science, Fuzhou, China). Images were taken immediately using an inverted fluorescence microscope (Carl Zeiss LSM 900, Oberkochen, Germany). The staining area and positive signals were quantified using Image J software.

### 2.10. Western Blot Analysis

Mouse aortas or VSMCs were homogenized in radio immunoprecipitation assay (RIPA) lysis buffer (Beyotime, Shanghai, China), to which was added 1% PMSF (Beyotime, Shanghai, China). Equal amounts of protein were separated by 8% or 10% SDS-PAGE and transferred to polyvinylidene fluoride membranes. After blocking non-specific sites with 5% BSA buffer, the membranes containing proteins were incubated overnight with primary antibodies at 4 °C, and with secondary antibodies for 1 h at room temperature. The bands were detected using a SuperSignal Chemiluminescence Substrate (Thermo Scientific, Waltham, MA, USA). Images were captured using the FluorChem E system (Protein Simple, San Jose, CA, USA). The band densities were quantified using ImageJ software and normalized to that of GAPDH.

### 2.11. Statistical Analysis

The results are expressed as the means ± standard deviations (SDs). The unpaired two-tailed Student’s *t*-test was used for the difference comparison of two groups, and one-way ANOVA for three or more groups, with GraphPad Prism 8.0. The differences were accepted as values of *p* < 0.05, * *p* < 0.05, ** *p* < 0.01, *** *p* < 0.001, and **** *p* < 0.0001.

## 3. Results

### 3.1. Effects of BHB on Body Weight and Food Intake of Mice

**Figure 1:**
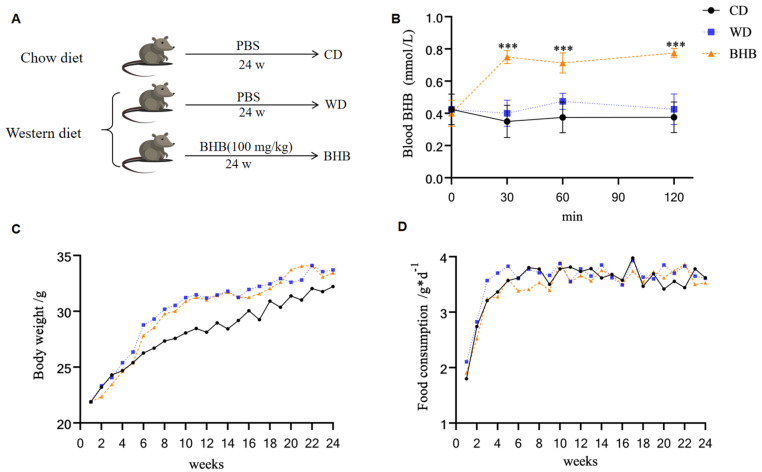
Effects of BHB on blood ketone level, body weight, and food consumption of mice. CD: chow diet group; WD: Western diet group; BHB: WD + BHB treatment group. (**A**) ApoE^−/−^ mice were fed with chow diet or Western diet, and gavaged with PBS or BHB (100 mg/kg) every day for 24 weeks; (**B**) the blood BHB level after gavage; (**C**) body weights of the abovementioned mice; (**D**) food intakes of the abovementioned mice; n = 6–8 in all data; *** *p* < 0.001.

Animals were grouped as shown in Figure 1A. To confirm that BHB treatment can increase blood BHB levels, tail blood was collected and measured immediately at baseline (time 0), before gavage, and at subsequent time points (30, 60, and 120 min). As shown in Figure 1B, BHB supplementation elevated blood BHB levels throughout the experiment (2 h). The prolonged effects of BHB on body weight and food consumption in mice were also evaluated. During the first 10 weeks, the body weight of the BHB group was slightly lower than that of the WD group, although the differences were not statistically significant. By week 11, the body weights of both groups ultimately reached the same level (Figure 1C). Similarly, over the first nine weeks, food consumption in the BHB group was lower than in the WD group but, again, there was no statistically significant difference (Figure 1D). These results indicate that BHB supplementation at this dosage has no obvious impact on body weight or food intake.

### 3.2. Impacts of BHB on Blood Glucose and Lipid Profiles

**Figure 2:**
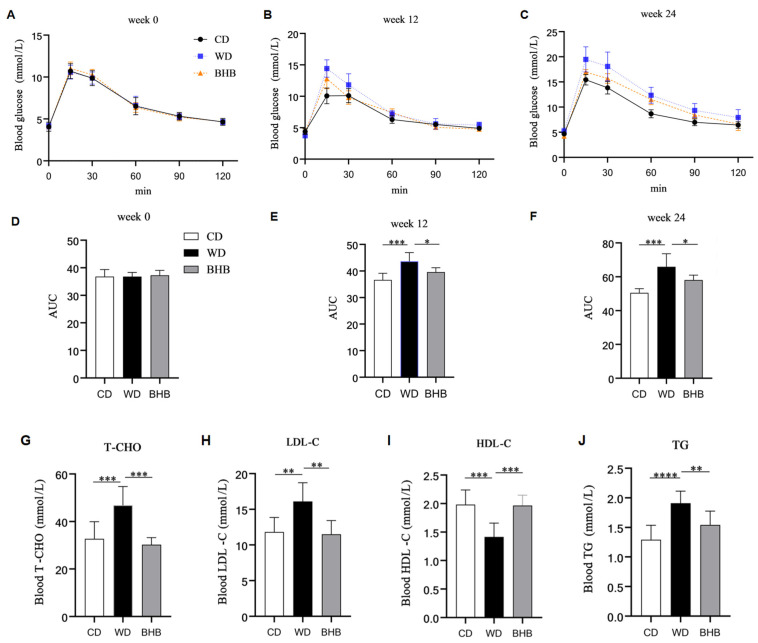
Effects of BHB on blood glucose and lipid profiles of mice. CD: chow diet group; WD: Western diet group; BHB: WD + BHB treatment group. OGTT results of the abovementioned mice at (**A**) week 0, (**B**) week 12, (**C**) week 24; and glucose AUC of the abovementioned mice at (**D**) week 0, (**E**) week 12, (**F**) week 24. (**G**) Blood TC level; (**H**) blood LDL-C level; (**I**) blood HDL-C level; (**J**) blood TG level; n = 6–8 in all data; * *p* < 0.05, ** *p* < 0.01, *** *p* < 0.001, and **** *p* < 0.0001.

Given that dyslipidemia and hyperglycemia are significant risk factors for AC, the effects of BHB supplementation on blood glucose and lipid levels were investigated. OGTTs were performed at weeks 0, 12, and 24 to assess changes in glucose tolerance of mice, and the serum lipid profiles were measured at week 24. The results show that glucose tolerance in the WD group declined at weeks 12 and 24 compared to the control group (Figure 2A–F). In contrast, BHB treatment improved the glycemic response in the mice. Furthermore, BHB treatment increased blood HDL-C levels while decreasing TC, LDL-C, and TG levels in the mice (Figure 2G–J). These findings suggest that BHB has beneficial effects on both glycemic control and lipid metabolism in model mice.

### 3.3. BHB Ameliorated Atherosclerotic Calcification in ApoE^−/−^ Mice

**Figure 3:**
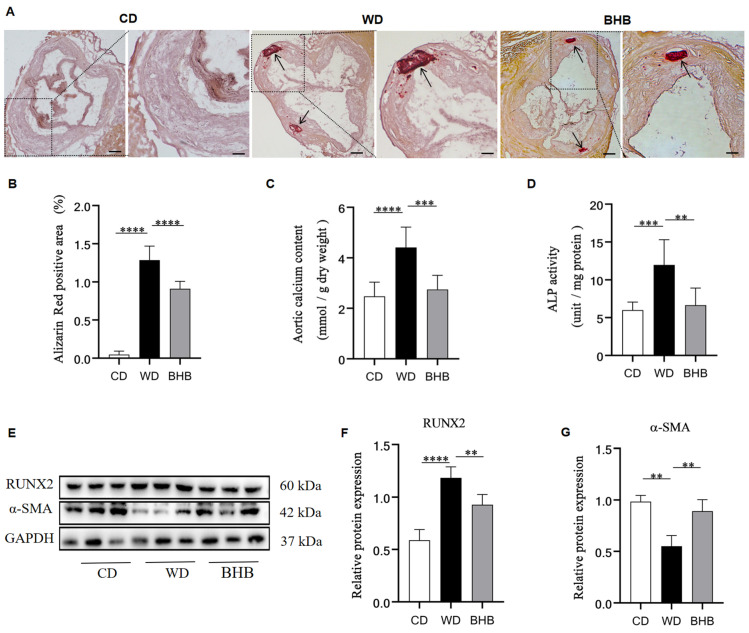
BHB inhibits diet-induced atherosclerotic calcification in ApoE^−/−^ mice. CD: chow diet group; WD: Western diet group; BHB: WD + BHB treatment group. (**A**) Typical pictures of the Alizarin Red S-stained aortic roots. The black arrows point to the calcified area (red); (**B**) calcification area quantification in aortic roots; (**C**) calcium concentration calculation in the aortas; (**D**) quantification of the ALP activity; (**E**–**G**) protein expression of RUNX2 and α-SMA; scale bar: 100 µm; n = 6–8 for each group; ** *p* < 0.01, *** *p* < 0.001, and **** *p* < 0.0001.

The effects of BHB on the calcification of atherosclerotic plaques were further assessed. Alizarin Red S staining revealed an elevated percentage of calcified area in the aortic roots of the WD group (Figure 3A). Additionally, the aortas of the model mice exhibited significantly higher calcium concentration compared to normal mice (Figure 3C). Furthermore, there was an increase in the activity of ALP, a hallmark of osteochondrogenic transition, in the aortas of model mice in the WD group (Figure 3D). Moreover, Western blot analysis revealed elevated expression of the osteogenic marker RUNX2 and decreased expression of the contractile marker α-SMA (Figure 3E–G). However, the aortas of the BHB group showed less calcium deposition and lower ALP activity than the WD group (Figure 3A–C). These mitigative effects of BHB were further confirmed by the elevation of α-SMA and the reduction of RUNX2 in the aortas (Figure 3E–G).

### 3.4. BHB Downregulates the Expression of the ERS Chaperone and Inhibits ERS-Mediated Apoptosis in Mouse Aortas

**Figure 4:**
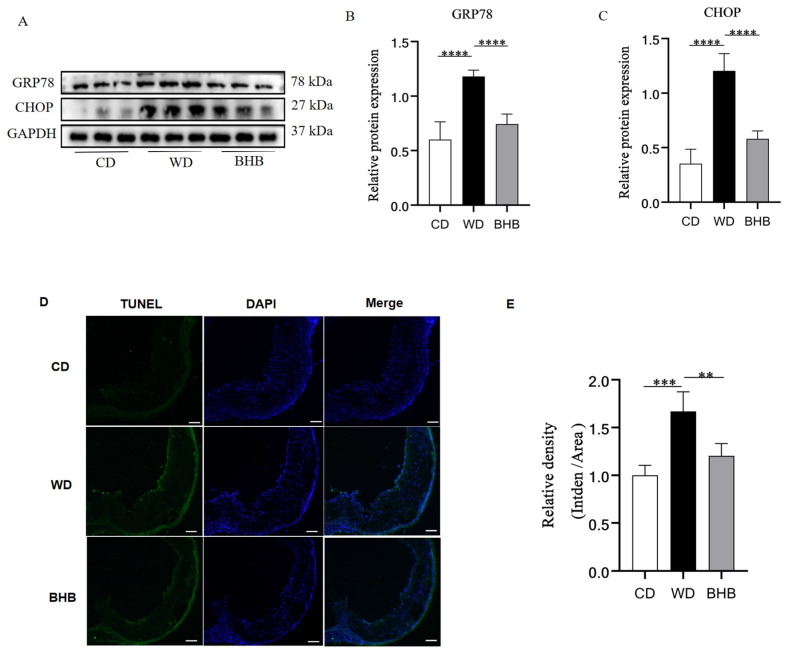
The levels of ERS marker, ERS-mediated apoptosis pathway expression, and apoptosis levels in mice aortas. CD: chow diet group; WD: Western diet group; BHB: WD + BHB treatment group. (**A**–**C**) Protein expression of GRP78 and CHOP in mice aortas; (**D**,**E**) representative immunofluorescence staining of TUNEL (green) in aortic roots. The color blue represents the nucleus of cells. Scale bar: 50 µm; n = 6–8 for each group; ** *p* < 0.01, *** *p* < 0.001, and **** *p* < 0.0001.

Since ERS is a major risk factor for vascular calcification, we further investigated whether BHB affects the ERS pathway in mice. Glucose-regulated protein 78 (GRP78) expression was first detected in mice aortas. Compared to the control group, GRP78 levels were elevated in the WD group, and BHB treatment reduced GRP78 protein expression by 31.9% (Figure 4A,B), indicating that BHB attenuates the ERS response in ACs. Next, the expression of C/EBP homologous protein (CHOP), a marker of the ERS-mediated apoptosis pathway, was analyzed. CHOP levels were significantly higher. In contrast, BHB treatment reduced CHOP expression by 51.6% compared to the WD group (Figure 4A,C). Moreover, the percentage of TUNEL-positive cells in the BHB group was considerably lower (by 27.8%) than in the WD group (Figure 4D).

### 3.5. BHB Attenuated Calcification of Rat VSMC

**Figure 5:**
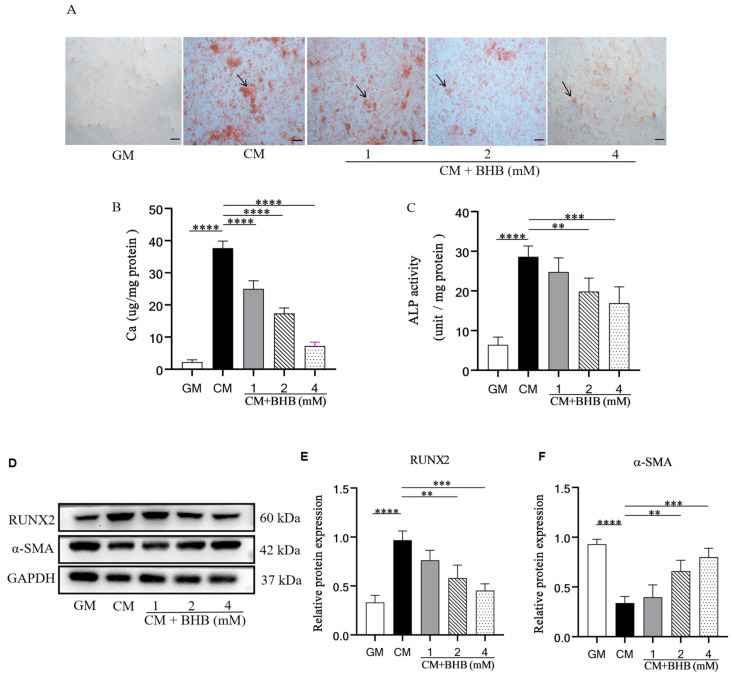
BHB inhibits the calcification of rat VSMC. GM: growth medium group; CM: calcification medium group; BHB: CM+ BHB (1, 2, and 4 mM) treatment group. (**A**) Typical pictures of VSMC stained with Alizarin Red S. The black arrows point to the calcium deposition area (red); (**B**) the calcium content of VSMC; (**C**) the ALP activity of VSMC; (**D**–**F**) protein expression of RUNX2 and α-SMA; scale bar: 100 µm; n = 3 for each group; ** *p* < 0.01, *** *p* < 0.001, and **** *p* < 0.0001.

To verify these results in mice, rat VSMCs, cultured in the CM with or without BHB for 7 days, were applied to observe the effects of BHB on VSMC calcification. Alizarin Red S staining revealed calcification nodules in the CM group but not in the GM group, and BHB dose-dependently ameliorated VSMC calcification in the CM group (Figure 5A). Furthermore, BHB treatment remarkably reduced the calcium content of the cells (Figure 5B). Moreover, the activity of ALP was also decreased by BHB treatment (Figure 5C). Moreover, in the presence of BHB, the level of RUNX2, an indicator of osteogenic transformation, was downregulated, while α-SMA, a marker of the normal contractile phenotype, was elevated (Figure 5D–F). These findings confirm that BHB can reduce calcium deposition and inhibit osteogenic differentiation of VSMCs.

### 3.6. BHB Downregulated the Expression of the ERS Chaperone and Inhibited ERS-Mediated Apoptosis in Rat VSMC

**Figure 6:**
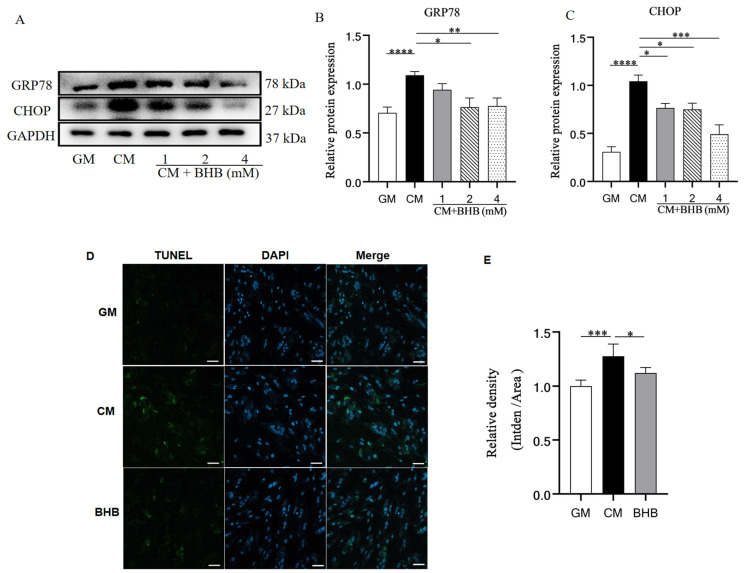
The levels of ERS marker, ERS-mediated apoptosis pathway expression, and apoptosis levels in rat VSMCs. GM: growth medium group; CM: calcification medium group; BHB: CM+ BHB (1, 2, and 4 mM) treatment group. (**A**–**C**) Western blot analysis of GRP78 and CHOP in mice; (**D**,**E**) representative immunofluorescence staining of TUNEL (green) in rat VSMCs (cells in the BHB group were cultured in CM with 4 mM BHB). The color blue represents the nucleus of cells. Scale bar: 50 µm; n = 6–8 for each group; * *p* < 0.05, ** *p* < 0.01, *** *p* < 0.001, and **** *p* < 0.0001.

Next, we further explored whether BHB could suppress ERS-mediated apoptosis in VSMCs. Western blot analysis showed that the GRP78 level in the CM group was higher than in the GM group but, in VSMCs treated with BHB, GRP78 expression was reduced (Figure 6A,B), indicating that BHB can attenuate the ERS response in VSMCs. We next examined the expression of CHOP, a crucial indicator of ERS-mediated apoptosis, to investigate whether BHB can inhibit ERS-mediated apoptosis in vitro. The CM group exhibited higher levels of CHOP expression. Furthermore, BHB treatment dramatically reduced CHOP expression (Figure 6A,C). Additionally, TUNEL staining showed that the CM group had a higher proportion of TUNEL-positive cells than the GM group. Nonetheless, BHB treatment greatly reduced the percentage of TUNEL-positive cells by 12.1% (Figure 6D,E). These findings show that BHB could attenuate VSMC calcification by restraining ERS-mediated apoptosis.

### 3.7. BHB Improves VC by Activating AMPK/Nrf2 Pathway

**Figure 7:**
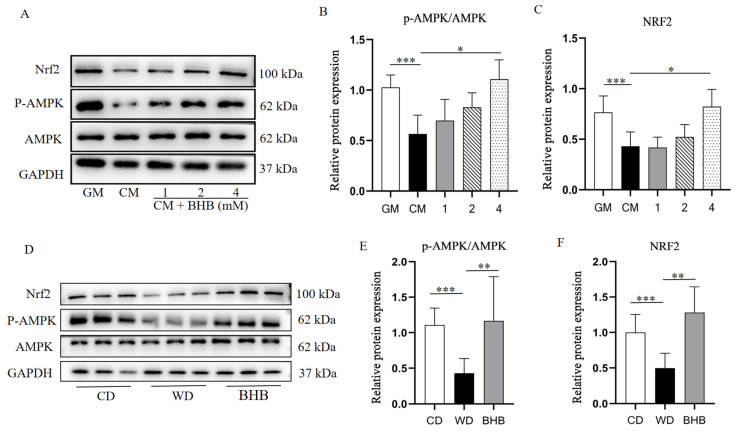
BHB treatment increased the protein level of p-AMPK/AMPK ratio and Nrf2 both in VSMCs and mice aortas. (**A**–**C**) Western blot analysis of p-AMPK, AMPK, and Nrf2 in VSMCs, cultured in GM, CM, and CM with BHB; GM: growth medium group; CM: calcification medium group; BHB: CM+ BHB (1, 2, and 4 mM) treatment group; n = 3 for each group; (**D**–**F**) Western blot analysis of p-AMPK, AMPK, and Nrf2 in mice aortas of the CD, WD, and BHB groups; CD: chow diet group; WD: Western diet group; BHB: WD + BHB treatment group; n = 6–8 for each group; * *p* < 0.05, ** *p* < 0.01, *** *p* < 0.001.

**Figure 8:**
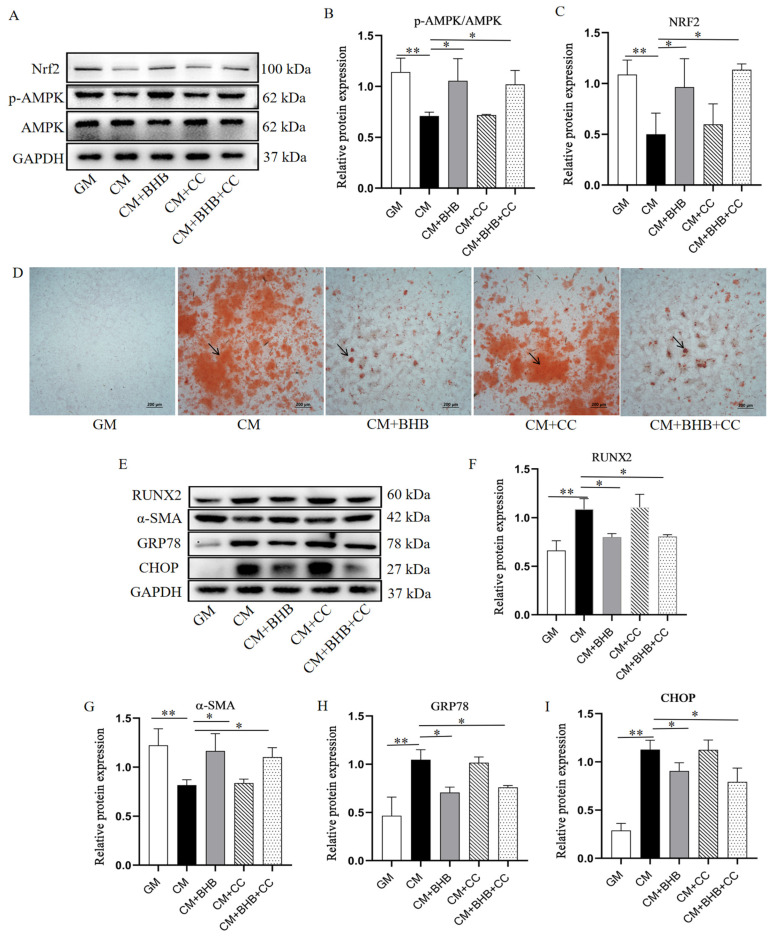
BHB mitigates VSMC calcification by activating AMPK/Nrf2 pathway. GM: growth medium group; CM: calcification medium group; CC: Compound C (cells treated with BHB were cultured in CM with 4 mM BHB); (**A**–**C**) Western blot analysis of p-AMPK, AMPK, and Nrf2 in VSMCs; (**D**) typical pictures of VSMCs stained with Alizarin Red S. The black arrows point to the calcium deposition area (red); (**E**–**I**) Western blot analysis of RUNX2, α-SMA, GRP78, and CHOP. Scale bar: 100 µm; n = 3 for each group; * *p* < 0.05, ** *p* < 0.01.

To further investigate the potential molecular mechanism, the protein expression of pAMPK, AMPK, and Nrf2 in the aortas of mice and VSMCs were detected. As shown in Figure 7, the protein levels of the p-AMPK/AMPK ratio and its downstream antioxidant protein Nrf2 were significantly lower in the WD group and in VSMCs cultured with CM, when compared to the normal control group. In contrast, BHB treatment significantly increased the protein expression of both the p-AMPK/AMPK ratio and Nrf2. To identify whether the AMPK pathway is involved in the effects of BHB, the AMPK pathway inhibitor Compound C (CC, 25 µM) was applied. As shown in Figure 8, the addition of CC inhibited the effects of BHB on the p-AMPK/AMPK ratio and the expression of Nrf2, and also reversed the effect of BHB on cell calcium deposition, as well as the protein expression of RUNX2, α-SMA, GRP78, and CHOP.

## 4. Discussion

BHB was reported to have beneficial effects on cardiovascular diseases [27]. However, the experiment duration of the BHB intervention in these studies is relatively short term, typically less than 6 months. Whether BHB can affect the progression of AC remains unclear. This study found that 24 weeks of BHB supplementation could suppress the calcification of atherosclerotic plaques in ApoE^−/−^ mice by alleviating ERS-mediated apoptosis of VSMCs. In addition, prolonged BHB supplementation improves glucose tolerance and blood lipid profiles in mice, although body weight and food intake do not change significantly. Thus, BHB may be a promising strategy for preventing and alleviating AC.

Vascular calcification is a common cardiovascular complication [28]. For calcification located in the medial vessel wall, BHB has been reported to suppress this process by enhancing autophagy [22] in VSMCs and downregulating histone deacetylase 9 (HDAC9) in rats with chronic kidney disease and C57BL/6J mice overloaded with vitamin D_3_ [25]. However, for intimal calcifications such as AC, the function of BHB is not clear. We hypothesized that BHB may have beneficial effects on AC. To test this hypothesis, we employed a commonly utilized mouse model to observe the long-term effect of BHB on AC and relative risk factors. The results showed that 24 weeks of BHB supplementation reduced the calcification area, calcium content, and ALP activity in the aortas of mice. Additionally, the osteochondrogenic transition of VSMCs in the aortas was inhibited by BHB. Since research has shown that ERS-mediated apoptosis is a major cause of vascular calcification [29,30], VSMC apoptosis can promote its osteoblastic phenotypic transformation [31]. We next explored whether BHB can suppress the ERS-mediated apoptosis in the aortas of mice. We measured the GRP78 and CHOP protein levels in mice aortas and detected cell apoptosis in aortic root sections. Interestingly, BHB decreased the GRP78 and CHOP expression, and reduced the percentage of apoptotic cells, indicating that ERS-mediated apoptosis was inhibited. These findings from mice experiments were further verified in rat VSMCs. A study reported that, in mice treated with BHB, hepatic ERS was suppressed as the GRP78 level decreased, but the CHOP protein level remained unchanged [32]. This discrepancy may be due to the fact that, in that study, mice were injected with BHB only once before sacrifice, and BHB can be quickly eliminated by the bloodstream. In contrast, in our study, mice were treated with BHB for 24 weeks, resulting in a reduction in both GRP78 and CHOP expression in the aorta, thus confirming the prolonged effects of BHB on ERS.

BHB was found to inhibit medial vascular calcification by downregulating HDAC9 [25], and to protect hepatocytes against ERS through the BHB receptor G-protein-coupled receptor 109a (Grp109a) [23,33]. Ten weeks of BHB supplementation can ameliorate atherosclerosis by stabilizing Ca^2+^ homeostasis in the endoplasmic reticulum and modulating the subsequent inflammation via Grp109a in ApoE^−/−^ mice [23]. However, the formation of atherosclerotic calcification is a longer and more complex pathological process, involving mechanisms distinct from those of medial calcification. Whether BHB can attenuate atherosclerotic calcification and inhibit the ERS-mediated apoptosis of VSMCs is still unknown. In this study, we selected the dosage of BHB based on previous research. However, the intervention duration was longer (24 weeks versus 10 weeks). In accordance with previous findings, after 24 weeks of BHB supplementation, we observed a suppression of atherosclerotic lesion calcification. Furthermore, ERS-mediated apoptosis in both the aortas of mice and VSMCs was also inhibited. Given the signal regulation ability of BHB, we speculate that BHB may inhibit ERS-mediated apoptosis by binding to its receptor and acting as a molecular regulator, thus impeding the progression of atherosclerotic calcification.

The activation of the AMPK/Nrf2 pathway has been linked to the prevention of ERS-mediated apoptosis [34,35]. What is more, AMPK activation can inhibit VSMC calcification. Mechanically, AMPK activation downregulates the expression and activity of RUNX2, inhibits ERS, and thereby suppresses the osteoblastic differentiation of VSMCs [36,37]. Furthermore, the activation of the Nrf2 pathway, mediated by heme, has been shown to attenuate the calcification of valve interstitial cells [38]. Importantly, research studies have reported that empagliflozin regulates the Nrf2 anti-inflammation pathway via AMPK activation, and could attenuate VC in CKD mice [39]. In view of the protective effect of the AMPK/Nrf2 pathway in VC, we investigated whether BHB could attenuate AC by activating this pathway. We found that, in the BHB intervention group, the expression of p-AMPK and Nrf2 were significantly elevated. Moreover, the addition of compound C, the AMPK pathway inhibitor, could inhibit the expression of Nrf2 and reverse the mitigative effect of BHB on VSMC calcification. These results indicate that BHB supplementation may alleviate AC by inhibiting the ERS-mediated apoptosis via activation the AMPK/Nrf2 pathway. Previous studies have reported that BHB mitigates chondrocyte senescence and apoptosis via the AMPK pathway [40], and BHB-induced Nrf2 activation mediated anti-senescent effects in HK-2 cells treated with H_2_O_2_ [41]. What is more, BHB dehydrogenase has been reported to mitigate diabetes-induced atherosclerosis through the activation of Nrf2 [42]. Together, these studies indicate that BHB may exert its health protective effects via regulating the AMPK/Nrf2 pathway.

Apart from maintaining the homeostasis of endoplasmic reticulum, the effects of BHB on the risk factors associated with atherosclerotic calcification were also evaluated. Overweight and obesity are closely linked to vascular calcification [43,44]. Therefore, this study explored the prolonged effects of BHB on body weight and food intake in mice, finding no significant changes. However, a previous study reported a significant decrease in body weight after 10 weeks of BHB supplementation. Due to variations in ketone sources, dosages, animal models, intervention time points, and durations, the effects of ketone supplementation on animal body weight are debatable [45,46,47]. In addition, the mice in our study may have developed tolerance to BHB after a few weeks of intervention; thus, the food consumption and body weight gradually reached the same levels, in our study. Based on these findings and previous studies, we speculate that short-term supplementation (less than 10 weeks) may produce more pronounced changes in body weight and appetite. In brief, the effects of ketone supplements on appetite and body weight are influenced by multiple factors, and further studies are needed to clarify these effects.

Hyperglycemia and hyperlipidemia also play certain roles in vascular calcification [48,49]. Many studies have reported that exogenous ketone consumption can temporarily lower blood glucose and decrease the mean daily glucose, but not fasting glucose [50,51,52,53]. Similarly, after treatment with BHB for 24 weeks, the fasting glucose level was not changed, but the peak value and AUC of the OGTT were decreased by BHB, which may be attributed to BHB’s ability to alleviate insulin resistance [53]. Consistent with previous research, our study revealed that lipid profiles were significantly improved in mice treated with BHB, and the underlying mechanisms include the activation of Grp109a [54], inhibition of the reverse cholesterol transport pathway [24], and suppression of adipocyte lipolysis [47]. However, there are discrepancies in the reported effects of BHB on lipid profiles across different studies. These differences may be attributed to variations in intervention duration, methods of administration, and dosages of BHB.

Our study also has several limitations. First, we only used rat VSMCs to further confirm our findings in mice due to experimental constraints. Although the methods and mechanisms to induce the calcification of rat, mouse, and human VSMCs are similar, it would be better to verify our results using additional cell models. Second, while our study indicates that BHB may inhibit AC by restraining the ERS-mediated apoptosis pathway via the AMPK/Nrf2 pathway, more research is needed to confirm the current findings and explore the underlying mechanisms in greater depth.

## 5. Conclusions

In summary, the present study demonstrates that BHB can alleviate atherosclerotic calcification by inhibiting ERS-mediated apoptosis via the AMPK/Nrf2 pathway. BHB may be an effective therapeutic target for treating AC.

## Data Availability

The original contributions presented in the study are included in the article, further inquiries can be directed to the corresponding author.

## References

[1] Libby P. (2021). The changing landscape of atherosclerosis. Nature.

[2] Jebari-Benslaiman S., Galicia-Garcia U., Larrea-Sebal A., Rekondo Olaetxea J., Alloza I., Vandenbroeck K., Benito-Vicente A., Martin C. (2022). Pathophysiology of Atherosclerosis. Int. J. Mol. Sci..

[3] Silvestre-Roig C., de Winther M.P., Weber C., Daemen M.J., Lutgens E., Soehnlein O. (2014). Atherosclerotic Plaque Destabilization Mechanisms, Models, and Therapeutic Strategies. Circ. Res..

[4] Huang H., Virmani R., Younis H., Burke A.P., Kamm R.D., Lee R.T. (2001). The impact of calcification on the biomechanical stability of atherosclerotic plaques. Circulation.

[5] Hutcheson J.D., Maldonado N., Aikawa E. (2014). Small entities with large impact: Microcalcifications and atherosclerotic plaque vulnerability. Curr. Opin. Lipidol..

[6] Durham A.L., Speer M.Y., Scatena M., Giachelli C.M., Shanahan C.M. (2018). Role of smooth muscle cells in vascular calcification: Implications in atherosclerosis and arterial stiffness. Cardiovasc. Res..

[7] Harman J.L., Jorgensen H.F. (2019). The role of smooth muscle cells in plaque stability: Therapeutic targeting potential. Br. J. Pharmacol..

[8] Grootaert M.O.J., Moulis M., Roth L., Martinet W., Vindis C., Bennett M.R., De Meyer G.R.Y. (2018). Vascular smooth muscle cell death, autophagy and senescence in atherosclerosis. Cardiovasc. Res..

[9] Yang M.-Y., Wang Y.-B., Han B., Yang B., Qiang Y.-W., Zhang Y., Wang Z., Huang X., Liu J., Chen Y.-D. (2018). Activation of aldehyde dehydrogenase 2 slows down the progression of atherosclerosis via attenuation of ER stress and apoptosis in smooth muscle cells. Acta Pharmacol. Sin..

[10] Ren J., Bi Y., Sowers J.R., Hetz C., Zhang Y. (2021). Endoplasmic reticulum stress and unfolded protein response in cardiovascular diseases. Nat. Rev. Cardiol..

[11] Song X., Li J., Jiao M., Chen Y., Pan K. (2021). Effect of endoplasmic reticulum stress-induced apoptosis in the role of periodontitis on vascular calcification in a rat model. J. Mol. Histol..

[12] Rao Z., Zheng Y., Xu L., Wang Z., Zhou Y., Chen M., Dong N., Cai Z., Li F. (2022). Endoplasmic Reticulum Stress and Pathogenesis of Vascular Calcification. Front. Cardiovasc. Med..

[13] Lu Y., Yuan T., Min X., Yuan Z., Cai Z. (2021). AMPK: Potential Therapeutic Target for Vascular Calcification. Front. Cardiovasc. Med..

[14] Saha S., Buttari B., Panieri E., Profumo E., Saso L. (2020). An Overview of Nrf2 Signaling Pathway and Its Role in Inflammation. Molecules.

[15] Xu W., Zhao T., Xiao H. (2020). The Implication of Oxidative Stress and AMPK-Nrf2 Antioxidative Signaling in Pneumonia Pathogenesis. Front. Endocrinol..

[16] Zimmermann K., Baldinger J., Mayerhofer B., Atanasov A.G., Dirsch V.M., Heiss E.H. (2015). Activated AMPK boosts the Nrf2/HO-1 signaling axis-A role for the unfolded protein response. Free. Radic. Biol. Med..

[17] Liu X., Xu Y., Cheng S., Zhou X., Zhou F., He P., Hu F., Zhang L., Chen Y., Jia Y. (2021). Geniposide Combined with Notoginsenoside R1 Attenuates Inflammation and Apoptosis in Atherosclerosis via the AMPK/mTOR/Nrf2 Signaling Pathway. Front. Pharmacol..

[18] Puchalska P., Crawford P.A. (2021). Metabolic and Signaling Roles of Ketone Bodies in Health and Disease. Annu. Rev. Nutr..

[19] Nielsen R., Moller N., Gormsen L.C., Tolbod L.P., Hansson N.H., Sorensen J., Harms H.J., Frokiaer J., Eiskjaer H., Jespersen N.R. (2019). Cardiovascular Effects of Treatment With the Ketone Body 3-Hydroxybutyrate in Chronic Heart Failure Patients. Circulation.

[20] Chakraborty S., Galla S., Cheng X., Yeo J.-Y., Mell B., Singh V., Yeoh B., Saha P., Mathew A.V., Vijay-Kumar M. (2018). Salt-Responsive Metabolite, β-Hydroxybutyrate, Attenuates Hypertension. Cell Rep..

[21] Krishnan M., Hwang J.S., Kim M., Kim Y.J., Seo J.H., Jung J., Ha E. (2020). β-hydroxybutyrate Impedes the Progression of Alzheimer’s Disease and Atherosclerosis in ApoE-Deficient Mice. Nutrients.

[22] Liang J., Huang J., He W., Shi G., Chen J., Huang H. (2021). β-Hydroxybutyric Inhibits Vascular Calcification via Autophagy Enhancement in Models Induced by High Phosphate. Front. Cardiovasc. Med..

[23] Zhang S.-J., Li Z.-H., Zhang Y.-D., Chen J., Li Y., Wu F.-Q., Wang W., Cui Z.J., Chen G.-Q. (2021). Ketone Body 3-Hydroxybutyrate Ameliorates Atherosclerosis via Receptor Gpr109a-Mediated Calcium Influx. Adv. Sci..

[24] Xu Z., Zhang M., Li X., Wang Y., Du R. (2022). Exercise Ameliorates Atherosclerosis via Up-Regulating Serum β-Hydroxybutyrate Levels. Int. J. Mol. Sci..

[25] Lan Z., Chen A., Li L., Ye Y., Liang Q., Dong Q., Wang S., Fu M., Li Y., Liu X. (2022). Downregulation of HDAC9 by the ketone metabolite β-hydroxybutyrate suppresses vascular calcification. J. Pathol..

[26] Dai X., Liu S., Cheng L., Huang T., Guo H., Wang D., Xia M., Ling W., Xiao Y. (2022). Epigenetic Upregulation of H19 and AMPK Inhibition Concurrently Contribute to S-Adenosylhomocysteine Hydrolase Deficiency-Promoted Atherosclerotic Calcification. Circ. Res..

[27] Yurista S.R., Chong C.-R., Badimon J.J., Kelly D.P., de Boer R.A., Westenbrink B.D. (2021). Therapeutic Potential of Ketone Bodies for Patients with Cardiovascular Disease *JACC* State-of-the-Art Review. J. Am. Coll. Cardiol..

[28] Sutton N.R., Malhotra R., St. Hilaire C., Aikawa E., Blumenthal R.S., Gackenbach G., Goyal P., Johnson A., Nigwekar S.U., Shanahan C.M. (2023). Molecular Mechanisms of Vascular Health: Insights From Vascular Aging and Calcification. Arterioscler. Thromb. Vasc. Biol..

[29] Duan X.-H., Chang J.-R., Zhang J., Zhang B.-H., Li Y.-L., Teng X., Zhu Y., Du J., Tang C.-S., Qi Y.-F. (2013). Activating transcription factor 4 is involved in endoplasmic reticulum stress-mediated apoptosis contributing to vascular calcification. Apoptosis.

[30] Duan X., Zhou Y., Teng X., Tang C., Qi Y. (2009). Endoplasmic reticulum stress-mediated apoptosis is activated in vascular calcification. Biochem. Biophys. Res. Commun..

[31] Li M., Wang Z.W., Fang L.J., Cheng S.Q., Wang X., Liu N.F. (2022). Programmed cell death in atherosclerosis and vascular calcification. Cell Death Dis..

[32] Tagawa R., Kawano Y., Minami A., Nishiumi S., Yano Y., Yoshida M., Kodama Y. (2019). β-hydroxybutyrate protects hepatocytes against endoplasmic reticulum stress in a sirtuin 1-independent manner. Arch. Biochem. Biophys..

[33] Bae H.R., Kim D.H., Park M.H., Lee B., Kim M.J., Lee E.K., Chung K.W., Kim S.M., Im D.S., Chung H.Y. (2016). β-Hydroxybutyrate suppresses inflammasome formation by ameliorating endoplasmic reticulum stress via AMPK activation. Oncotarget.

[34] Zhang Y., Liu Y., Liu X., Yuan X., Xiang M., Liu J., Zhang L., Zhu S., Lu J., Tang Q. (2022). Exercise and Metformin Intervention Prevents Lipotoxicity-Induced Hepatocyte Apoptosis by Alleviating Oxidative and ER Stress and Activating the AMPK/Nrf2/HO-1 Signaling Pathway in db/db Mice. Oxidative Med. Cell. Longev..

[35] Hou X., Fu M., Cheng B., Kang Y., Xie D. (2019). Galanthamine improves myocardial ischemia-reperfusion-induced cardiac dysfunction, endoplasmic reticulum stress-related apoptosis, and myocardial fibrosis by suppressing AMPK/Nrf2 pathway in rats. Ann. Transl. Med..

[36] Cao X., Li H., Tao H., Wu N., Yu L., Zhang D., Lu X., Zhu J., Lu Z., Zhu Q. (2013). Metformin Inhibits Vascular Calcification in Female Rat Aortic Smooth Muscle Cells via the AMPK-eNOS-NO Pathway. Endocrinology.

[37] Li K.-X., Du Q., Wang H.-P., Sun H.-J. (2019). Death-associated protein kinase 3 deficiency alleviates vascular calcification via AMPK-mediated inhibition of endoplasmic reticulum stress. Eur. J. Pharmacol..

[38] Balogh E., Chowdhury A., Ababneh H., Csiki D.M., Toth A., Jeney V. (2021). Heme-Mediated Activation of the Nrf2/HO-1 Axis Attenuates Calcification of Valve Interstitial Cells. Biomedicines.

[39] Lu C.-W., Lee C.-J., Hsieh Y.-J., Hsu B.-G. (2023). Empagliflozin Attenuates Vascular Calcification in Mice with Chronic Kidney Disease by Regulating the NFR2/HO-1 Anti-Inflammatory Pathway through AMPK Activation. Int. J. Mol. Sci..

[40] Zhuang H., Ren X., Zhang Y., Li H., Zhou P. (2024). β-Hydroxybutyrate enhances chondrocyte mitophagy and reduces cartilage degeneration in osteoarthritis via the HCAR2/AMPK/PINK1/Parkin pathway. Aging Cell.

[41] Kim M.N., Moon J.H., Cho Y.M. (2021). Sodium-glucose cotransporter-2 inhibition reduces cellular senescence in the diabetic kidney by promoting ketone body-induced NRF2 activation. Diabetes Obes. Metab..

[42] Lin J., Ren Q., Zhang F., Gui J., Xiang X., Wan Q. (2023). D-β-Hydroxybutyrate Dehydrogenase Mitigates Diabetes-Induced Atherosclerosis through the Activation of Nrf2. Thromb. Haemost..

[43] Huang Y., Ruan Z., Lin W., Chen Z., Zhang L., Li Z. (2022). Association Between Weight Change and Increased Likelihood of Abdominal Aortic Calcification Among Men. J. Endocr. Soc..

[44] Kowall B., Lehmann N., Mahabadi A.A., Moebus S., Erbel R., Joeckel K.H., Stang A. (2019). Associations of metabolically healthy obesity with prevalence and progression of coronary artery calcification: Results from the Heinz Nixdorf Recall Cohort Study. Nutr. Metab. Cardiovasc. Dis..

[45] Davis R.A.H., Deemer S.E., Bergeron J.M., Little J.T., Warren J.L., Fisher G., Smith D.L., Fontaine K.R., Dickinson S.L., Allison D.B. (2019). Dietary *R*,*S*-1,3-butanediol diacetoacetate reduces body weight and adiposity in obese mice fed a high-fat diet. Faseb. J..

[46] Stubbs B.J., Cox P.J., Evans R.D., Cyranka M., Clarke K., de Wet H. (2018). A Ketone Ester Drink Lowers Human Ghrelin and Appetite. Obesity.

[47] Caminhotto R.d.O., Medeiros Komino A.C., Silva F.d.F., Andreotti S., Laurato Sertie R.A., Reis G.B., Lima F.B. (2017). Oral β-hydroxybutyrate increases ketonemia, decreases visceral adipocyte volume and improves serum lipid profile in Wistar rats. Nutr. Metab..

[48] Suryavanshi S.V., Kulkarni Y.A. (2017). NF-κβ: A Potential Target in the Management of Vascular Complications of Diabetes. Front. Pharmacol..

[49] Tintut Y., Hsu J.J., Demer L.L. (2018). Lipoproteins in Cardiovascular Calcification: Potential Targets and Challenges. Front. Cardiovasc. Med..

[50] Ari C., Murdun C., Koutnik A.P., Goldhagen C.R., Rogers C., Park C., Bharwani S., Diamond D.M., Kindy M.S., D’Agostino D.P. (2019). Exogenous Ketones Lower Blood Glucose Level in Rested and Exercised Rodent Models. Nutrients.

[51] Myette-Cote E., Caldwell H.G., Ainslie P.N., Clarke K., Little J.P. (2019). A ketone monoester drink reduces the glycemic response to an oral glucose challenge in individuals with obesity: A randomized trial. Am. J. Clin. Nutr..

[52] Falkenhain K., Daraei A., Forbes S.C., Little J.P. (2022). Effects of Exogenous Ketone Supplementation on Blood Glucose: A Systematic Review and Meta-analysis. Adv. Nutr..

[53] Chung J.Y., Kim O.Y., Song J. (2022). Role of ketone bodies in diabetes-induced dementia: Sirtuins, insulin resistance, synaptic plasticity, mitochondrial dysfunction, and neurotransmitter. Nutr. Rev..

[54] Plaisance E.P., Lukasova M., Offermanns S., Zhang Y., Cao G., Judd R.L. (2009). Niacin stimulates adiponectin secretion through the GPR109A receptor. Am. J. Physiol.-Endocrinol. Metab..

